# Decoy receptors as biomarkers for exploring aetiology and designing new therapies

**DOI:** 10.1093/ckj/sfae222

**Published:** 2024-07-18

**Authors:** Carmine Zoccali, Giovanni Tripepi, Vianda Stel, Edouard L Fu, Francesca Mallamaci, Friedo Dekker, Kitty J Jager

**Affiliations:** Renal Research Institute, NY, USA; Institute of Molecular Biology and Genetics (Biogem), Ariano Irpino, Italy; Associazione Ipertensione Nefrologia Trapianto Renale (IPNET), c/o Nefrologia, Grande Ospedale Metropolitano, Reggio Calabria, Italy; CNR-IFC, Institute of Clinical Physiology, Research Unit of Clinical Epidemiology, Reggio Calabria, Italy; ERA Registry, Amsterdam UMC location and the University of Amsterdam, Department of Medical Informatics, Amsterdam, the Netherlands; Amsterdam Public Health, Quality of Care, Amsterdam, the Netherlands; Fresenius Medical Care, Global Medical Office, Crema, Italy; CNR-IFC, Institute of Clinical Physiology, Research Unit of Clinical Epidemiology, Reggio Calabria, Italy; Department of Clinical Epidemiology, Leiden University Medical Center, Leiden, the Netherlands; Fresenius Medical Care, Global Medical Office, Crema, Italy; Department of Clinical Epidemiology, Leiden University Medical Center, Leiden, the Netherlands; Nephrology, Dialysis and Transplantation Unit, Azienda Ospedaliera “Bianchi-Melacrino-Morelli” Grande Ospedale Metropolitano of Reggio Calabria, Italy

**Keywords:** biomarkers, CKD, decoy receptors, epidemiology, inflammation

## Abstract

Soluble decoy receptors (DR) are circulating proteins that act as molecular traps for ligands that modulate various signalling pathways. These proteins can be exploited as biomarkers and, in some cases, as drugs in various disease contexts. Inflammation is a key area where DRs have shown significant potential. By binding to pro-inflammatory cytokines, inflammatory DRs, such as soluble tumour necrosis factor receptors (sTNFRs), can inhibit downstream inflammatory signalling. This modulation of the inflammatory response holds promise for therapeutic interventions in various inflammatory conditions, including cardiovascular and chronic kidney diseases. Soluble DRs for advanced glycation end products (sRAGE) bind to advanced glycation end products (AGEs), reducing their detrimental effects on vascular function and atherosclerosis. High circulating sRAGE levels are associated with a lower risk for CV events, highlighting the potential of these soluble receptors for assessing the role of AGEs in CV diseases and managing the attendant risk. DRs may serve as biomarkers and therapeutic agents to advance our understanding of disease mechanisms and improve patients' outcomes. Their ability to modulate signalling pathways in a controlled manner opens up new opportunities for therapeutic interventions in various diseases, ranging from inflammation to cardiovascular and renal disorders.

Decoy receptors (DRs), a class of proteins with wide-ranging effects, have emerged as significant regulators of cellular signalling pathways, particularly inflammation signalling [1], with implications for a broad spectrum of biological processes and diseases.

Beyond the classical concept of the ligand as a soluble molecule and the receptor as a membrane-bound protein, different forms of ligands and receptors exist on the cell surface and in the extracellular space [2]. These include membrane receptors and soluble receptors, both in ligand-bound and ligand-free form. Ligands can be soluble, unassociated, bound to membrane receptors or soluble receptors in the extracellular space or circulation. Soluble receptors primarily bind to their ligands and neutralize them. The term ‘decoy receptor’ originates from the concept of a decoy, which is something used to distract or mislead. In the context of receptors, a DR functions by binding to ligands and preventing them from interacting with functional receptors, thereby diverting, or neutralizing their effects. This mechanism helps regulate the signalling pathways activated by the ligands, preventing them from interacting with membrane-bound receptors and influencing cellular responses (Fig. 1B). Some receptors need a membrane co-receptor to signal (Fig. 1D). Several examples of these co-receptors exist. For example, CD40 is a co-receptor that interacts with DRs TNFα-R1 and R2. DRs ‘TNFα-related apoptosis-inducing ligands’ (TRAIL)-R1 and TRAIL-R2 are co-receptors that interact to regulate the apoptotic signalling of TNF ligands. IL-1R1 is a co-receptor that interacts with the DR IL-1R2 to modulate the signalling of IL-1β (IL-1). VEGFR-2 is a co-receptor that interacts with DR VEGFR-1 to modulate the signalling of vascular endothelial growth factor (VEGF) and regulate angiogenesis and tumour growth. Like membrane receptors, co-receptors can also be released in soluble forms and bind to receptors or ligands on the cell surface or in the extracellular space.

**Figure 1:**
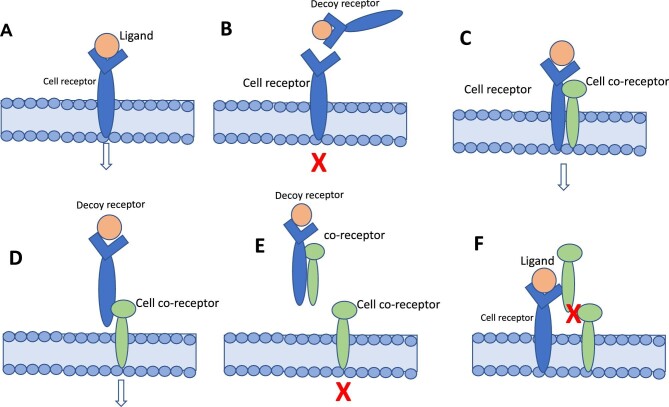
(**A**) Cell receptor and its ligand at the cell surface. The link of the ligand to the corresponding receptor activates cell signalling. (**B**) A DR ‘captures’ the ligand in the circulation and prevents it from activating the cell receptor. (**C**) Some receptors need to receptors to activate cell signalling. (**D**) Without a cell receptor, the DR–ligand complex can still activate cell signalling by linking to a co-receptor. (**E**) If a circulating co-receptor binds to the DR–ligand complex in the circulation, it may prevent this complex from activating cell signalling via the cell co-receptor. (**F**) The soluble, circulating co-receptor, can bind to the cell receptor, and this link may prevent cell signalling if the link with a cell co-receptor is needed to activate cell signalling. The arrow indicates normal cell signalling, and the red X indicates the cell signalling block.

Soluble receptors, derived from membrane receptors, are generated through (i) proteolytic cleavage, (ii) alternative mRNA splicing, or (iii) release in extracellular vehicles such as exosomes [1]. The first mechanism is realized via enzymes belonging to the family of A Disintegrin and Metalloproteases (ADAMS), ADAM17 (or TACE—TNF-alpha converting enzyme), and ADAM10 being the most relevant among these proteolytic enzymes. The second mechanism involves alternative splicing of a membrane receptor mRNA that omits the receptor's transmembrane domain. Like other secreted proteins, the resultant soluble receptor is secreted from the cell into the extracellular space. As to the third mechanism, cytokines and growth factor receptors have been identified in extracellular vesicles (EVs), such as exosomes, maintaining full-length receptors on the vesicle membrane. These released receptors, including TNFR1 and IL-6R, can bind ligands to modulate signalling properties. TNFR1 EVs lack an active signalling complex but can bind exogenous TNF. Functional proteases in exosomes suggest the potential shedding of receptor parts, although cytokine receptor shedding from exosomes has not been demonstrated yet.

The binding properties of ligands, receptors, co-receptors, and adhesion molecules determine the signalling outcome. SDRs can also interact with other cell membrane proteins, adding to their complexity (Table 1). In this regard, soluble adhesion molecules modulate inflammation by regulating the recruitment and activation of immune cells. Indeed, selectins, integrins, and immunoglobulin superfamily members are released into the bloodstream in response to inflammation and can either promote or inhibit the inflammatory response [3]. For almost every membrane receptor of cytokines, growth factors, and cell adhesins, soluble versions are naturally produced by cells. Moreover, viruses hijacked these same DRs during their co-evolution with their hosts [4].

**Table 1:** This structured summary provides an overview of the cell membrane proteins that DRs can interact with, the nature of their interactions, and the resulting biological effects.

**Cell membrane proteins**	**Interaction with decoy receptors**	**Biological effects**
Receptor Tyrosine Kinases (RTKs)	Competing for ligand binding or forming heterodimers with RTKs	Modulation of cell growth, survival, and differentiation pathways
G Protein-Coupled Receptors (GPCRs)	Sequestering ligands or forming complexes with GPCRs	Regulation of GPCR-mediated signalling events, impacting neurotransmission, hormone signalling, and immune responses
Adhesion Molecules	Interacting with adhesion molecules	Influencing cell adhesion, migration, and tissue organization
Immune Checkpoint Proteins	Engaging with immune checkpoint proteins	Modulation of T cell activation, immune surveillance, and immune evasion mechanisms
Receptor-associated Signalling Complexes	Interacting with components of signalling complexes	Disruption of signal transduction pathways and alteration of cellular responses

**Table 2:** Examples of DRs discussed in this review (see also main text).

**DR**	**Pathophysiology and prognosis**	**Therapeutic potential**
Inflammation DRs		
IL-1R2	Binds to IL-1 cytokines, regulates inflammation in cardiovascular diseases	Anakinra used for autoimmune diseases, with potential impact in heart failure
sTNFRs	Compete with TNF-α receptors, predict CKD progression and cardiovascular events	Etanercept approved for rheumatoid arthritis, biosimilars available
TRAIL DRs	Involved in apoptosis, lower levels linked to cardiovascular diseases	Promising in cancer treatment, no studies in cardiovascular or kidney diseases
DR for IL-6	Binds to IL-6, associated with increased cardiovascular risk	Research ongoing for potential treatment in inflammatory diseases
DcR1	Binds to TRAIL ligand, regulates apoptosis pathways	Potential role in cancer therapy
DcR2	Binds to TRAIL ligand, modulates apoptosis signalling pathways	Investigated for cancer treatment
DcR3	Binds to Fas ligand and LIGHT, regulates immune responses and cell death	Implicated in autoimmune diseases, potential therapeutic target
DR for IL-17	Binds to IL-17 cytokines, regulates inflammation and immune responses	Investigated for autoimmune diseases and inflammatory conditions
Cardiovascular DRs		
DR for VEGF	Binds to VEGF, modulates angiogenesis and vascular permeability	Potential target in cancer therapy and retinal vascular diseases
RAGE (Receptor for Advanced Glycation End Products)	Binds to advanced glycation end products, implicated in inflammation and vascular dysfunction	Potential target for diabetic complications and cardiovascular diseases
DR for PDGF	Binds to PDGF, regulates cell growth and proliferation in cardiovascular diseases	Investigated for potential use in vascular remodelling and fibrosis

As alluded to before, the effect of DRs may also depend on the presence of co-receptors and other interactions [1]. Figure 1C and d schematize the receptor–co-receptor interaction. The soluble nature of DRs allows for easy quantification in biological fluids, such as blood or urine, making them exploitable for diagnostic and prognostic purposes. Their presence and activity can provide insights into specific signalling pathways and aid disease diagnosis and prognosis. In addition, these soluble receptors offer opportunities to develop targeted therapies.

A unique property of some SDRs is that they mimic the effect of an intervention countering the ligand they block. In this respect, a DR of a noxious ligand would be expected to be inversely related to disease outcomes because it limits the exposure of the corresponding tissue receptor to the noxious ligand. However, most DRs affecting inflammation signalling that are measurable in biological fluids are directly rather than inversely associated with organ damage and the risk of adverse health events, as is the case for most DRs of the inflammatory system [1]. This apparently paradoxical relationship depends on their complex interaction with co-receptors and on the fact that some inflammatory DRs have pleiotropic effects and serve to modulate rather than abolish the inflammatory response. Examples of pleiotropic effects of DRs include Decoy Receptor 3 (DcR3) [5], a receptor binding the Fas ligand; a receptor expressed by T lymphocytes, and the ‘Lymphotoxin-like, exhibiting Inducible expression, and competing with Herpes Virus (HSV) Glycoprotein D’ (LIGHT), an entry mediator for HSV regulating both immune responses and cell death. Similarly, the DR for ‘Tumour Necrosis Factor-Related Apoptosis-Inducing Ligand’ (TRAIL) binds to TRAIL, Fas ligand, and ‘TNF-related weak inducer of apoptosis’ (TWEAK) [5], a ligand regulating apoptosis and immune responses.

As remarked, some receptors need co-receptors to function, and soluble co-receptors may either activate or inhibit cell signalling, as depicted in Fig. 1E and F.

Overall, with some notable exceptions, most circulating DRs that interfere with inflammatory mechanisms are direct, rather than inverse, predictors of health outcomes.

Herein, we will discuss a series of inflammatory, cardiovascular (CV), and metabolic DRs, focusing on their relationship with disease status and the risk of clinical events and, in specific cases, underlining their therapeutic potential.

## DECOY RECEPTORS AFFECTING INFLAMMATION

### Interleukin 1 receptor type II (IL-1R2)

#### Pathophysiology and prognosis

IL-1R2 binds to interleukin-1α, interleukin-1β, and interleukin-1 receptor antagonist (IL-1Ra), preventing their binding to conventional receptors and subsequent signalling. IL-1R2 acts as a DR on the cell surface or in a soluble form (sIL-1R2) in circulation, inhibiting the IL-1β-mediated inflammatory response [6]. IL-1R1 is a receptor that functions as a co-receptor, interacting with the DR IL-1R2, which helps regulate the effects of IL-1β. This interaction between IL-1R1 and IL-1R2 is important for fine-tuning the immune response and maintaining immune balance. Maintaining a balance between agonist and antagonist levels avoids exaggerated inflammatory responses. IL-1β plays a central role as a mediator propagating the inflammatory response and is implicated in atherothrombosis. For this reason, IL-R2 and other receptors functionally linked to IL-1β are expected to be recruited following an inflammatory event such as myocardial infarction. In a study by Orrem *et al.* [7] sIL-1R2, IL-1Ra, and sIL-1R1 levels were elevated after myocardial infarction. sIL-1R2 levels correlated positively with C-reactive protein, myocardial infarct size, and change in indexed left ventricular end-diastolic and end-systolic volume acutely, and, after 4 months, negatively with LV ejection fraction. Patients with levels of sIL-1R2 above the median value in the acute phase were more likely to develop LV dilatation. Thus, because these receptors are shed as a compensatory response to inflammation, their circulating levels are directly, rather than inversely, associated with heart disease and do not capture their mitigating effect on inflammation in myocardial infarction. Unquestionably, IL-1β, a main ligand of the IL1-R2 receptor, exerts adverse effects on the CV system. Indeed, the Canakinumab Anti-inflammatory Thrombosis Outcomes Study showed that blocking inflammation with the anti-IL-1β monoclonal antibody canakinumab reduced heart attacks, strokes, and new-onset diabetes among patients with prior myocardial infarction [8]. Furthermore, in the same trial, this treatment reduced the risk of incident lung cancer and lung cancer mortality [9]. The situation is different in some renal diseases. Indeed, higher sIL-2R was independently associated with renal recovery in a series of patients with the autoimmune tubule-interstitial disease and, on discrimination analysis, this DR had a good area under the receiver operator curve (80%) for predicting a favourable outcome [10]. In line with a favourable action of high sIL1-R in kidney diseases, high levels of the soluble IL-1 receptor antagonist (sIL-1Ra) predicted progression towards kidney failure in patients with IgA nephropathy [11].

#### Therapeutic potential

Anakinra (Kineret, Swedish Orphan Biovitrum AB, Stockholm, Sweden) is a recombinant form of the IL-1R antagonist that inhibits the activity of IL-1β and has been primarily used as a treatment for autoimmune diseases that involve dysregulated IL-1 signalling, such as rheumatoid arthritis [12]. In a systematic review that included eight anakinra clinical trials in patients with heart failure, the use of the drug was associated with a reduction in C-reactive protein (CRP) levels, indicating anti-inflammatory effects. However, these trials failed to show meaningful effects of the drug on cardiac function and exercise capacity or adverse events [13]. In the ACTION trial—a pilot, multicentre, randomized, placebo-controlled trial of an IL-1 receptor antagonist, anakinra—the median plasma concentrations of high sensitivity CRP from baseline to week 24 was 41% in the anakinra group and 6% in the placebo group, documenting a relevant anti-inflammatory effect of the drug in these patients [14]. Anakinra's effect needs to be tested in well-powered trials in this population.

### Soluble tumour necrosis factor receptors (sTNFRs)

#### Pathophysiology and prognosis

TNF-α is critical to immune system responses [15]. Inflammatory and stress response pathways are activated when TNF-α binds to TNF receptors (TNFRs). Two main TNF-α receptors exist: type 1 (TNFR1) and type 2 (TNFR2) [15]. CD40 is a co-receptor that interacts with DRs TNFR1 an TNFR2 to modulate the signalling of TNFα family cytokines. Similarly, TNF-related apoptosis-inducing ligands (TRAIL)-R1 and TRAIL-R2 are co-receptors that interact with DRs TRAIL-R3 and TRAIL-R4 to regulate the apoptotic signalling of TNF ligands.

At the tissue level, TNFR1 is ubiquitously expressed and is a death receptor inducing apoptosis, whereas TNFR2 is mainly expressed in T cells, endothelial cells, and fibroblasts and stimulates NFκB signalling [15]. Both contain transmembrane domains but can also be cleaved by ADAM metallopeptidase domain 17 into soluble receptors (sTNFR1 and sTNFR2). sTNFR1 and sTNFR2 compete with the transmembrane forms by binding circulating TNF-α to inhibit/modulate the effects of this cytokine.

In rats treated with streptozocin, TNFα and IL-1β exert damaging effects at the kidney level by mediating the effect of advanced glycosylation end products [16]. However, soluble TNFRs are direct predictors of the progression of CKD in patients with type 1 and 2 diabetes [17]. Similarly, sTNFR1 and sTNFR2 concentration increases after myocardial infarction and correlates directly with infarct size and left ventricular end-diastolic volume and inversely with left ventricular ejection fraction [18] and are direct predictors of a composite endpoint, including death, recurrent myocardial infarction, stroke, or hospitalization for heart failure within 24 months after myocardial infarction [18].

#### Therapeutic potential

Etanercept (Enbrel, Wyeth Pharmaceuticals) is a fusion protein composed of a soluble TNF-alpha receptor issued by biotechnology. In the United States, etanercept has been approved by the Food and Drug Administration since 1998 to treat rheumatoid arthritis, showing an inadequate response to prior therapy with other disease-modifying antirheumatic drugs. Other marketing authorizations have been obtained in ankylosing spondylitis, polyarticular-course juvenile rheumatoid arthritis, and the treatment of psoriatic arthritis. The etanercept patent expired in the EU in 2015, and some etanercept copies have reached the production phase and are being used. In a recent meta-analysis, etanercept biosimilars were as effective and safe as the originator [19].

### DRs for Tumour Necrosis Factor-Related Apoptosis-Inducing Ligand (TRAIL)

#### Pathophysiology and prognosis

TRAIL is a member of the tumour necrosis factor superfamily [20]. It is expressed as a transmembrane protein on various cell types' cell surfaces or released as a soluble protein. One important characteristic of TRAIL is its ability to induce apoptosis (cell death) in cancer cells [20].

TRAIL binds to DRs TRAIL-R3/DcR1 and R4/DcR2 and Osteoprotegerin, showing a complex system with various biological effects. The anti-cancer activity of this signalling pathway is well-known [21]. Recent studies focused on its role in CV diseases [22]. TRAIL and its receptors are expressed in endothelial cells, vascular smooth muscle cells, and inflammatory cells. Studies suggest that TRAIL is involved in CV diseases, with lower levels linked to acute myocardial infarction and poor prognosis in patients with coronary artery disease or heart failure [23]. Low TRAIL levels are also associated with atheromatosis plaque formation in patients with chronic kidney disease [24]. Lower TRAIL levels increase the risk of death in patients with prevalent CV disease, with higher mortality rates linked to the lowest TRAIL levels [25].

#### Therapeutic potential

TRAIL-based therapy shows promise in cancer treatment by targeting cancer cells while sparing normal cells [21]. However, resistance to TRAIL in colorectal cancer proved to be a significant issue. No studies with TRAIL have been performed on CV and kidney diseases in humans.

### Decoy receptor for IL6

IL-6 is one of the most potent inflammatory cytokines implicated in various pathophysiological responses and diseases, from the response to infectious agents to CV, kidney, rheumatic, and cancer [26].

#### Pathophysiology and prognosis

One example of a DR for IL-6 is the soluble IL-6 receptor (sIL-6R), which can bind to IL-6 and block its signalling pathway. Elevated levels of sIL-6R are associated with an increased risk of CV diseases such as heart attack and stroke. This is because sIL-6R is involved in the inflammatory processes that contribute to the development of atherosclerosis, a condition where plaque builds up in the arteries and can lead to heart disease. In a large population-based case-control study in Stockholm, individuals in the highest quartile of sIL-6R had 40% higher risk of myocardial infarction [27]. Since the binary IL6: sIL6R complex is inactivated by sgp130 through the formation of the ternary IL6: sIL6R: sgp130 complex, in a subsequent study in the same cohort by the same investigators tested whether the ratio between the binary and ternary complexes (IL6:sIL6R to IL6:sIL6R:sgp130 ratio) associates with incident CV events [28]. In line with the hypothesis that active binary complexes are the main determinant of high CV risk, this study showed that individuals with a high ratio of circulating IL-6/sIL-6R to IL-6/IL-6R/gp130 complexes had an increased risk of disease, including coronary heart disease, sudden cardiac death, and ischaemic stroke. Levels of soluble IL6 receptors may be elevated in patients with kidney disease, potentially serving as a biomarker for disease progression [29] but no prospective cohort studies investigating the link between sILR and the progression of CKD or the risk of kidney failure have been produced so far.

#### Therapeutic potential

DRs for IL-6 are being researched as potential treatment options for diseases involving inflammation, like rheumatoid arthritis, inflammatory bowel disease, and certain cancers. Scientists are exploring ways to use DRs to regulate the IL-6 pathway and develop new therapies for these conditions. EVs are natural nanoparticles that can be modified to carry therapeutic molecules to specific targets. By engineering EVs to express IL6 signal transducer (IL6ST) DRs, researchers could selectively block the IL6 trans-signalling pathway while leaving the IL6 classical signalling pathway unaffected [30]. In studies using muscle cells and a mouse model of Duchenne muscular dystrophy (DMD), IL6ST DR EVs successfully inhibited the activation of STAT3, a protein involved in inflammation and muscle degeneration [30]. This study highlights the potential of targeting the IL6ST DR EVs as a potential therapy beyond rare muscle diseases.

### Decoy receptor 3 (DcR3)

#### Pathophysiology and prognosis 

DR 3, also known as Fas, is a protein encoded by the *FAS* gene in humans. This receptor is a cell death receptor that causes programmed cell death (apoptosis). Its soluble form can neutralize the biological functions of three members of the tumour necrosis factor superfamily (TNFSF): Fas ligand (FasL), LIGHT, and TL1A, which is a direct predictor of inflammatory disease progression and cancer metastasis [31]. Thus, this soluble DR indicates disease severity and does not capture this molecule's inherent protective effects on inflammatory mechanisms. Studies in CV disease go along with those with cancer, showing that DcR3 levels are raised in myocardial infarction and associated with the severity of inflammation in this disease [32].

#### Therapeutic potential 

DcR3 may have the potential to be a therapeutic target for various diseases, including cancer and autoimmune disorders. Some studies have explored the use of DcR3 as a drug target in preclinical models, with promising results in terms of modulating immune responses and reducing inflammation [5]. However, to date, no studies are testing this DR for the prevention and/or treatment of cardiovascular and renal diseases.

### Decoy receptor for Interleukin-17 (IL-17)

The interleukin-17 (IL-17) family, consisting of six members, plays a crucial role in inflammation, immunity, and disease progression. IL-17 involves various biological functions, including inflammatory responses during infections and autoimmune diseases and enhancing protective immunity against pathogens. Despite its importance, the exact mechanisms by which IL-17 operates are still debated [33].

#### Pathophysiology and prediction 

DRs for IL17 can help regulate the immune response and reduce inflammation in conditions such as autoimmune diseases (e.g. rheumatoid arthritis, psoriasis), inflammatory bowel disease, and certain types of cancer. By binding to IL17 and preventing it from signalling through its regular receptors, DRs can help dampen the inflammatory response and alleviate symptoms associated with these conditions. There are still no studies about the prognostic value of these DRs for cardiovascular and renal diseases.

#### Therapeutic potential

DRs are a therapeutic agent that can bind to specific cytokines and prevent them from interacting with their natural receptors. This can reduce the cytokine's signalling activity, which can be beneficial in diseases where the cytokine is overactive or contributes to pathology. IL-17 (interleukin-17) is a pro-inflammatory cytokine that plays a key role in the immune response, particularly in the defence against fungal and bacterial infections. However, dysregulation of IL-17 signalling is implicated in various inflammatory and autoimmune diseases, such as psoriasis, ankylosing spondylitis, and rheumatoid arthritis.

In theory, DRs for IL-17, such as IL-17RA and IL-17RC, can treat these diseases by binding to IL-17 and preventing it from interacting with its natural receptors, thus inhibiting the IL-17-mediated inflammatory response. Two randomized clinical trials showed that secukinumab, a monoclonal antibody that targets IL-17A, mitigates psoriasis severity [34]. Another IL-17A inhibitor, ixekizumab, improved symptoms of ankylosing spondylitis in patients with this disease in two double-blind randomized trials [35]. While these studies focus on monoclonal antibodies that target IL-17A directly rather than DRs, they illustrate the therapeutic potential of modulating IL-17 activity in treating inflammatory diseases.

### G protein-coupled decoy chemokine receptors

These receptors, formerly known as DARC (Duffy antigen receptor for chemokines) and D6, act as ‘sinks’ for circulating chemokines in the body. They bind to chemokines in the bloodstream, preventing them from interacting with their target receptors on immune cells and thus regulating the levels of chemokines in the body [36]. This process helps to control the immune response and inflammation. The role of these DRs in various diseases and conditions, including inflammation, cancer, and infectious diseases, is being actively investigated [37].

## CARDIOVASCULAR AND METABOLIC DECOY RECEPTORS

### Vascular Endothelial Growth Factor Receptor-1 (VEGFR-1)

#### Pathophysiology and prognosis

EGFR-1 is a kinase-impaired tyrosine kinase receptor that negatively modulates angiogenesis by acting as a DR of VEGF, a fundamental regulator of both physiological and pathological angiogenesis [38]. The decoy characteristic of VEGFR-1 (also named fms‐like tyrosine kinase 1 [Flt‐1]) is required for normal development and angiogenesis [38]. VEGFR-2 is a co-receptor that interacts with DR VEGFR-1 to modulate the signalling of VEGF and regulate angiogenesis and tumour growth and another crucial factor coordinating angiogenesis with cardiomyocyte growth [39].

The relationship of these DRs with CV events is complex and incompletely understood. Low levels of VEGFR2 are independently associated with CV death and all-cause death in patients with chronic HF [40]. Since VEGF per se probably plays a favourable role in heart failure [41], such an inverse association suggests that low circulating levels of this receptor reflect a reduced representation of the same receptors at the myocardial level [41] rather than antagonism/capture of circulating VEGF. By contrast, high rather than low VEGFR1 and VEGFR2 predict a high risk of death in patients with acute coronary syndromes [42]. This effect, opposite to that registered in heart failure, is attributed to the fact that these DR are synthesized following cardiac ischaemia and mainly reflect an ongoing inflammatory process.

The kidney is a highly vascularized organ, and damage to the microvascular structure in the kidney is implicated in CKD progression. VEGF expression and signalling in the kidney are reduced in some experimental models, like in a model of ADPKD [43], and the administration of VEGF is protective in the same model [44]. However, antagonism of VEGF by its endogenous inhibitor (soluble VEGFR-1/sFlt-1) in preeclampsia and monoclonal antibody against VEGF in cancer patients causes proteinuria and renal dysfunction [44]. VEGF and its receptors are upregulated in other experimental animals and human type 1 and 2 diabetes, and inhibition of VEGF limits functional and structural alterations of the kidney in models of diabetic nephropathy [45], suggesting a deleterious role for VEGF in the pathophysiology of diabetic nephropathy [46]. Furthermore, deleterious effects of VEGF administration have been demonstrated in experimental sepsis and, conversely, a beneficial effect of VEGF-R1 in the same model [47]. Overall, the effects of VEGF and its receptors, both tissue-level receptors and soluble receptors, are model and disease-dependent, and the soluble DR of VEGF have no real potential for application in clinical and epidemiologic studies looking at CV [48] or kidney [49] outcomes in CKD.

#### Therapeutical potential

Aflibercept (Eylea), a DR for VEGF, is used to treat eye conditions like macular degeneration and diabetic retinopathy [50]. However, no specific intervention studies on CV and kidney diseases have been performed with this or similar drugs. In diabetic patients with macular disease receiving intravitreal anti-VEGF treatment, the eGFR declined during treatment, and the decline was maximal at the high and low extremes of the eGFR distribution in the study population. Patients with low baseline eGFR tended to require dialysis after P anti-VEGF treatment [51]. These findings clearly indicate that antagonizing VEGF with its DR exerts noxious kidney effects.

### Soluble receptors of advanced glycosylation end products (sRAGE)

#### Pathophysiology and prognosis

sRAGE inhibit the binding of AGEs to their receptors, deterring the inflammatory response that generally follows the activation of this receptor. sRAGEs are of utmost interest because circulating levels of this biomarker are inversely associated with coronary heart disease in nondiabetic men [52] as well as with atherosclerosis [53] and left ventricular hypertrophy [54] in CKD patients, thereby providing circumstantial evidence that countering AGEs may translate into beneficial CV effects in patients with coronary heart disease and CKD patients with CV disease. Indeed, these studies indicate that AGEs are noxious to the CV system [55] and, in a way, mimic clinical trials testing compounds that block the noxious effects of AGEs.

#### Therapeutic potential

A study conducted at two separate research centres found that the short-term administration of an antagonist to sRAGE protected against diabetes in mice [56]. This treatment with sRAGE led to an increase in regulatory T cells (Tregs) within various key areas such as the islets, pancreatic lymph nodes, and spleen, and Tregs ultimately resulted in enhanced insulin expression and function within the islets, thereby contributing to the overall protection against diabetes [56]. These findings suggest that targeting the RAGE receptor with sRAGE could be a potential therapeutic strategy for managing diabetes. Furthermore, sRAGE exerted an anti-atherogenic effect by blocking the activation of the RAGE signalling pathway induced by disturbed blood flow in a mouse model of partial carotid artery ligation [57]. AGEs have various effects on cell damage that can lead to neurological disorders. They interact with RAGE receptors, which activate intracellular signalling and lead to the expression of pro-inflammatory factors and cytokines [58]. This inflammatory cascade is linked to diseases such as Alzheimer's, traumatic brain injury, ALS, diabetic neuropathy, diabetes, and atherosclerosis. Imbalances in gut microbiota and intestinal inflammation are also connected to endothelial dysfunction, disrupted BBB, and the development of neurological diseases [58].

AGE antagonists manifested favourable effects in various experimental models [50]. In mice, sRAGE acts as a scavenger, blocking the corresponding membrane-bound receptor activation and inhibiting cardiac fibroblast differentiation and age-dependent cardiac fibrosis [59].

Inhibiting the interactions between AGE and RAGE with small molecule-based therapeutics can prevent the inflammatory events linked to these interactions, slowing down disease progression. RAGE antagonists such as Azeliragon [60] are being developed for treating neurological diseases like AD, but as of now, there are no FDA-approved therapeutics based on sRAGE or RAGE antagonists.

### DRs for Platelet-Derived Growth Factor (PDGF)

#### Pathophysiology and prognosis

PDGF receptors are cell surface tyrosine kinase receptors that bind to PDGF proteins. There are two main types of PDGF receptor, PDGFR-α and PDGFR-β, which play important roles in cell growth, development, and wound healing [61]. These receptors are often targeted in cancer therapy due to their involvement in cell proliferation and survival [61].

DRs of PDGF play a crucial role in CV disease by modulating the signalling pathways of PDGF and its receptors [61]. In CV diseases such as atherosclerosis and restenosis, the overactivation of PDGF signalling can lead to excessive smooth muscle cell proliferation and migration, contributing to the progression of the disease.

#### Therapeutic potential

DRs for PDGF, such as soluble forms of PDGFR-α and PDGFR-β, can potentially be used as therapeutic agents in CV disease. By blocking PDGF signalling, DRs may help to inhibit smooth muscle cell proliferation, reduce inflammation, and ultimately improve outcomes in patients with CV conditions. Specific anti-PDGF and anti-PDGFR molecules can also be designed, such as soluble receptors, aptamers, or oligonucleotides, but these potential interventions for the prevention and treatment of CV and renal disease remain to be explored [62].

## AN OVERVIEW

Overall, soluble DR are interesting biomarkers because, in some cases, they may help to dissect the causal implication of their ligand in human diseases. However, perhaps except sRAGE, they often show relationships with clinical outcomes difficult to interpret, a phenomenon depending on the counter-regulatory increase of their synthesis in high-risk conditions and, at least for some of these, on their pleiotropic effect that goes beyond their ‘decoy’ action.

Historically, observational epidemiology has only been able to infer causality. However, biological markers such as some DRs may allow a more refined view of the exposure-disease continuum and help the design of new drugs.

## PERSPECTIVE

Exploring inflammatory DRs, including IL-1R2 and sTNFRs, highlights their significance as biomarkers and modulators in disease progression. Their expression in myocardial infarction and renal diseases underscores the complexity of their role in inflammation and tissue injury. These receptors exhibit a nuanced relationship with clinical outcomes, suggesting their potential as targets for therapeutic intervention.

In CV and metabolic disorders, DRs such as VEGFR-1 and sRAGE command attention due to their impact on disease pathogenesis. The dichotomy of their effects in heart failure, acute coronary syndromes, and CKD emphasizes the context-dependent nature of their function. These findings indicate the necessity for precise interpretation of DR levels in various disease settings.

In conclusion, DR represent a unique class of biomarkers that can neutralize ligands and modulate signalling pathways, offering valuable insights into disease processes and potential therapeutic targets. While the relationships between DR and clinical outcomes can be complex, further research and understanding of their intricate roles will contribute to improved disease prognostication and the development of targeted interventions. Harnessing the full potential of DR as biomarkers holds promise for advancing precision medicine and improving patient outcomes.
